# Dynamic Neural Network Reconfiguration During the Generation and Reinstatement of Mnemonic Representations

**DOI:** 10.3389/fnhum.2018.00292

**Published:** 2018-07-20

**Authors:** Aiden E. G. F. Arnold, Arne D. Ekstrom, Giuseppe Iaria

**Affiliations:** ^1^Department of Psychology, Hotchkiss Brain Institute and Alberta Children’s Hospital Research Institute, University of Calgary, Calgary, AB, Canada; ^2^Center for Neuroscience, University of California, Davis, Davis, CA, United States; ^3^Department of Psychology, University of California, Davis, Davis, CA, United States; ^4^Neuroscience Graduate Group, University of California, Davis, Davis, CA, United States

**Keywords:** fMRI, graph theory, hippocampus, navigation, orientation, virtual environment

## Abstract

Mnemonic representations allow humans to re-experience the past or simulate future scenarios by integrating episodic features from memory. Theoretical models posit that mnemonic representations require dynamic processing between neural indexes in the hippocampus and areas of the cortex providing specialized information processing. However, it remains unknown whether global and local network topology varies as information is encoded into a mnemonic representation and subsequently reinstated. Here, we investigated the dynamic nature of memory networks while a representation of a virtual city is generated and reinstated during mental simulations. We find that the brain reconfigures from a state of heightened integration when encoding demands are highest, to a state of localized processing once representations are formed. This reconfiguration is associated with changes in hippocampal centrality at the intra- and inter-module level, decreasing its role as a connector hub between modules and within a hippocampal neighborhood as encoding demands lessen. During mental simulations, we found increased levels of hippocampal centrality within its local neighborhood coupled with decreased functional interactions between other regions of the neighborhood during highly vivid simulations, suggesting that information flow vis-à-vis the hippocampus is critical for high fidelity recapitulation of mnemonic representations.

## Introduction

One of the most striking features of the human mind is our ability to re-experience the past in vivid detail. Memories pervade daily life, allowing us to develop a sense of self, find new and familiar locations, and identify more effective strategies for interacting with the world. The ability to encode and reinstate complex mnemonic representations by binding features from previous experiences is thought to be the primary function of an episodic memory system in humans (Tulving, 2002). These representations are hypothesized to be conjunctive in nature, integrating sensory features from the environment – such as people, places, and objects – into holistic representations that can be used to guide behavior into the future (Davachi, 2006; Byrne et al., 2007; Chersi and Burgess, 2015). Theoretical and computational models suggest that these mnemonic representations are formed by integrating information processed throughout the neocortex in convergence zones (Marr, 1971; Damasio, 1989; Nadel and Moscovitch, 1997; Burgess, 2008; Meyer and Damasio, 2009), most notably the hippocampus (HC), where processes such as pattern completion allow representations to then be reinstated using a partial set of input features (Marr, 1971; Norman and O’Reilly, 2003; Rugg and Vilberg, 2013). Recent research has provided support for these models, showing that inter-regional communication dynamics assist to concentrate information flow to the HC (Mišić et al., 2014), and that the HC acts as a primary convergence zone during associative memory tasks (Gordon A.M. et al., 2014; Backus et al., 2016), allowing different types of information processed in the neocortex to be reinstated and integrated into a holistic representation (Staresina et al., 2013; Horner et al., 2015).

Although there is preliminary empirical evidence for hippocampal-based information integration during memory retrieval (Gordon A.M. et al., 2014; Iaria et al., 2014; Robin et al., 2014; Schedlbauer et al., 2014; Horner et al., 2015; Backus et al., 2016), key questions remain about how mnemonic representations are encoded across networks of brain regions. A critical but untested component of prominent theoretical models is that when encoding occurs, there is a heightened demand to integrate information processed in sensory and first-order association cortices into neural patterns within memory structures that form the basis of a mnemonic representation (Marr, 1971; Damasio, 1989; Squire and Zola-Morgan, 1991; McClelland et al., 1995; Nadel and Moscovitch, 1997; Meyer and Damasio, 2009). Recent work using functional magnetic resonance imaging (fMRI) analyses has shown that encoding associations between sensory features depends on neural activity in areas of the cortex specialized to the specific feature, which are encoded by neural indexes in the hippocampus (Horner et al., 2015), and that hippocampal–cortical functional interactions increase when stimuli features need to be combined into a single associative representation during retrieval (Zeithamova et al., 2012; Staresina et al., 2013; Gordon A.M. et al., 2014). This suggests that memory structures such as the hippocampus interact dynamically with other regions across the cortex during the initial encoding and subsequent reinstatement of a mnemonic representation. Surprisingly though, there has yet to be a systematic evaluation of global and local network topology during encoding using complex network measures such as graph theory. Graph theory allows for the quantification of more nuanced aspects of network processes (Bassett et al., 2012), specifically relating to communication dynamics and the integration of information across components of a network (Sporns et al., 2007; Bullmore and Sporns, 2009) that are of critical importance to understanding memory function in humans (Chrastil, 2012; Ekstrom et al., 2014). The central aim of this study is to provide such an investigation by quantifying putative changes in network topology and the dynamic role of the hippocampus within brain networks.

An important characteristic of brain network topology is modularity (Bertolero et al., 2015). Modular systems are sub-networks or communities defined by dense interconnections between intra-module components, with sparse or weak inter-module connections (Newman, 2006). Of importance here, the dynamic formation and interaction of modules and their components defined using functional interactions between brain regions has been proposed to provide a neural correlate for adaptability (i.e., learning) in the brain (Ghosh et al., 2008; Meunier et al., 2010; Werner, 2010), putatively through a reduced cost to rapidly change network configurations in response to environmental demands (Kirschner and Gerhart, 1998; Kashtan and Alon, 2005). Dynamic shifts in modularity have been associated with motor learning tasks (Bassett et al., 2011) and working memory paradigms such as the n-back task (Stanley et al., 2014; Cohen and D’Esposito, 2016). In the context of memory function, this view suggests that changes to the modularity of networks may allow the brain to regulate the degree to which sensory information is integrated into a neural index during encoding by altering the degree to which network modules communicate with one another. In the present study, we use this perspective on network modularity to investigate whether the dynamic reconfiguration of modular systems across the brain is associated with encoding and reinstating mnemonic representations based on the degree to which environmental features needs to be integrated over time. This tests the long held, but sparsely tested, perspective that information is integrated across sensory and associative cortices during representation encoding, and that this integration is mediated in part by the hippocampus (Nadel et al., 2000).

Drawing from theoretical models and empirical work, it is possible to formulate three key predictions about the basis of network reconfiguration and adaptability as mnemonic representations are encoded. First, when encoding demands are highest, the topology of brain networks should be organized in a manner that increases the capacity to integrate information processed across distributed systems in the brain. We term this the *global integration* hypothesis. Second, once representations are formed, there should be a reconfiguration of network topology from a state of global network integration to one of localized processing, as the need to integrate stimuli features lessens and the demand to reinstate and maintain neural representations within memory systems increases. We term this the *state transition* hypothesis. Third, critical convergence zones such as the HC should display flexibility in how they interact with global and local brain networks, such that when encoding demands are the highest, they act to integrate information across different systems in the brain, but change to localized processing as environmental feature integration demands decrease. We term this the *node flexibility* hypothesis.

Integrative processes additionally appear to play a role in the subsequent reinstatement and use of multi-featural representations (Gordon A.M. et al., 2014; Iaria et al., 2014; Robin et al., 2014; Schedlbauer et al., 2014; Horner et al., 2015; Backus et al., 2016). Prospection, the cognitive ability to think about, predict, and simulate possible future events is theorized to rely on a similar neurocognitive system dedicated to dynamically encoding experiences, extracting features from those memories, and actively combining those features into representations, or mental ‘scenes,’ that are used to optimize behavior (Buckner and Carroll, 2007; Schacter and Addis, 2007; Hassabis and Maguire, 2009; Moulton and Kosslyn, 2009; Schacter et al., 2012; Szpunar et al., 2013). As with the encoding of mnemonic representations, the HC is predicted to be critical to prospection, using a neural index to reinstate mnemonic representations through interactions with sensory and associative regions across the brain (Janzen and van Turennout, 2004; Horner et al., 2015). In humans, recent work has shown that goal-specific trajectories and intervening locations can be decoded during prospection using patterns of hippocampal activity (Brown et al., 2016), further supporting the role of the HC in coordinating the neural codes underlying the spatial context for prospective mental simulations. Importantly, however, prospective representations in humans appears to involve additional brain regions, including the parahippocampal, perirhinal, and retrosplenial cortices (Brown et al., 2016), suggesting that integrative and distributed information processes are also involved.

Although it appears that the HC dynamically interacts with other regions across the brain during prospection (Hassabis and Maguire, 2009; Schacter et al., 2012), fundamental questions remain about how this occurs and whether these interactions vary based on how featural information encoded in memory is integrated into a mnemonic representation subserving prospection. Variability in the fidelity of prospective mental simulations has been proposed as a measure of feature integration (Arnold et al., 2016), providing a quantifiable metric of how task-oriented mnemonic representations are generated through multi-feature reinstatement. Critically, this suggests that variance in the fidelity of prospective mental simulations may be associated with the degree of functional interactions within the hippocampal–cortical networks supporting prospection, rather than relying on neural processes occurring in the hippocampus alone, and may provide a window into how dynamic network processes support memory function.

As with encoding, it is possible to formulate specific research questions about how distributed and dynamic network processes provide a mechanism for prospection. First, we asked which regions of the brain coordinated information processing during mental simulations with the HC. Due to the putative role of the HC in coordinating the reinstatement of information processing in sensory and associative regions (Nadel et al., 2000; Horner et al., 2015; Backus et al., 2016), we hypothesized that simulations with high visual and spatial fidelity would be associated with increased functional connectivity between the HC and areas of the visual cortex, allowing for reinstatement of patterns of activity coding visual and spatial features of the route being simulated. We term this the *feature reinstatement* hypothesis. Second, we asked whether variance in the vividness and spatial coherence of mental simulations is associated with differences in hippocampal network topology. The capacity for both whole brain networks and the HC to efficiently coordinate information flow has been shown to facilitate the reinstatement of spatial contexts during retrieval tasks (Arnold et al., 2014b; Schedlbauer et al., 2014). This suggests that feature reinstatement and integration during prospection may also benefit from network topologies with increased communication efficiency, as features and their associative structure are recapitulated across the brain and integrated into a task-oriented mnemonic representation used to simulate behavior. Based on this perspective, we hypothesized that high simulation fidelity would require more widespread interactions between the HC and neocortex, and therefore demonstrate increases in graph theoretical measures quantifying the communication capacity of hippocampal networks. We term this the *feature integration* hypothesis.

**FIGURE 1:**
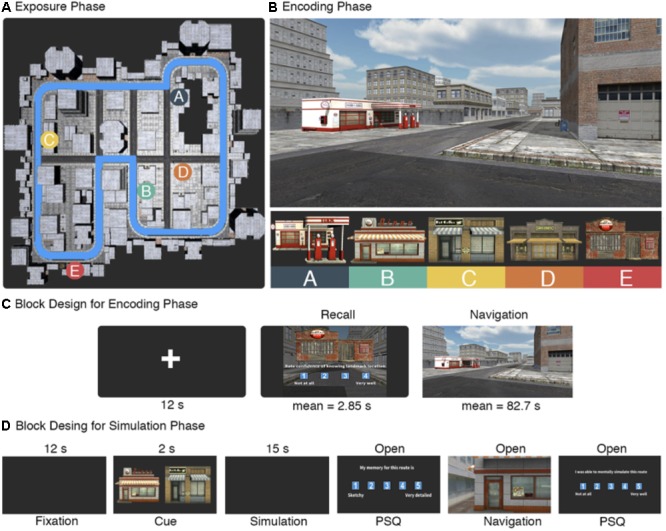
Task design. **(A)** Top-down view of the city. During the exposure phase, participants viewed a video of passive first-person movement along the city perimeter outlined in blue. Also displayed are the locations of the five target landmarks. **(B)** During the encoding phase, participants navigated between the five target landmarks. The city was composed of buildings using variations of three architectural styles, while the target landmarks were selected to be visually salient. **(C)** Block sequence order during scanning of the encoding phase. Each trial began with a fixation cross, followed by a recall block in which one of the target landmarks was displayed and participants rated on a scale of 1–4 their confidence in knowing its location. A navigation block followed, where participants were instructed to navigate to the landmark as quick as possible. **(D)** Timing information for the simulation phase. After the initial 12 s fixation period, participants were cued with a starting landmark (left) and a target landmark (right). They were given 15 s to simulate movement between the two landmarks in as much detail as possible. Completion of the PS-Q was open ended as was the subsequent navigation period between the two cued landmarks and the two remaining questions of the PS-Q.

To test the three outlined hypotheses on encoding mnemonic representations and the two on reinstating representations during prospection, we constructed a large-scale virtual city and calculated bivariate and graph theoretical measures using fMRI data acquired while people encoded the spatial layout of landmarks in the city and then conducted mental simulations of the different routes between landmarks. Spatial navigation is a model system for understanding how mnemonic representations are encoded and reinstated to guide behavior and inform decision making processes (Chersi and Burgess, 2015; Arnold et al., 2016), with past research demonstrating that the topology of resting-state (Arnold et al., 2014b) and task-active networks facilitate the accurate reinstatement of spatial representations (Watrous et al., 2013; Arnold et al., 2014a; Schedlbauer et al., 2014). **Figure 1** provides an overview of the task. The analyses herein were conducted on the navigation blocks from the encoding phase, and the simulation blocks from the simulation phase. For the *global integration* hypothesis during representation encoding, we calculated the modularity index Q, global efficiency, and global flow of distributed networks, and compared navigation blocks from the encoding phase where participants were unsure about landmark locations to trials in which they were highly confident of knowing the landmark location. Similarly, for the *node flexibility* hypothesis, we calculated and compared the betweenness centrality, participation, and flow coefficient for the HC between high and low confidence navigation blocks in the encoding phase. These graph theoretical metrics assess the centrality of the HC at the global, inter-module, and local network level, respectively. Third, for the *state transition* hypothesis, we calculated the local efficiency of the hippocampal networks during navigation blocks of the encoding phase to identify whether localized network processing increased as the need to integrate environmental information decreased. For prospection, we tested the *feature reinstatement* hypothesis on simulation blocks from the simulation phase using general psychophysiological (gPPI) models to identify areas of the brain showing increased functional connectivity with the HC during prospection based on high fidelity representations. For the *feature integration* hypothesis, we again used the node-based graph theoretical measures of betweenness centrality, flow coefficient, and local efficiency using hippocampal nodes to identify potential differences in communication efficiency relating to variance in representation fidelity observed during the simulation blocks of the simulation phase.

## Materials and Methods

### Participants

Fourteen right-handed undergraduates (nine female; mean age = 21.64, *SD* = 2.56) recruited through the University of Calgary research participation pool participated in the study and the study was approved by the research ethics board at the university (CHERB 22848). Sample size was similar to previous studies on the function of memory networks (Ekstrom and Bookheimer, 2007; Libby et al., 2012; Watrous et al., 2013; Arnold et al., 2014b). Participants were pre-screened to exclude anyone who reported previously experiencing nausea while playing a videogame. All participants provided informed consent prior to scanning, received $50 reimbursement whether they completed the experiment or not, and were debriefed after the experiment.

### Environment Design

The virtual city was designed using Unity3D (version 4.6^1^). The city was composed of an interconnecting series of roads lined with buildings (**Figure 1A**). The configuration of the roads was constructed to be nearly symmetric across the city to minimize the potential to derive location information based on global geometrical cues. The city buildings consisted of target landmarks and non-target buildings. The non-target buildings were variations of three architectural styles that were repeated throughout the city and selected to be visually similar in order to reduce their use as spatial cues during navigation trials. Five target landmarks were selected to be visually unique relative to the rest of the city (**Figure 1B**). The location of the target landmarks was selected by applying a 10×10 grid over the city layout and randomly selecting grid locations to place the landmarks. Numerous shortcuts were created by placing walkable paths between the buildings and a series of back alleyways. We also included two blockades on the main roads in order to ensure that the shortest path between any two target landmarks were only available by taking shortcuts. Movement speed was capped at 6 virtual m/s, which approximates to a 4.47× increase over an average real world walking speed of 1.34 m/s given the relative scale of the virtual city. Post-experiment interviews suggested that participants primarily used relative orientation of target landmarks to one another to guide navigation. Aside from the exposure phase (see below), participants viewed the city by projecting it on a mirror in the scanner and moved using four buttons coded to forward and backward movement, and left and right rotation.

### Task Design

The task was divided into three phases: an exposure, encoding, and simulation phase. Prior to entering the scanner, participants were given an overview of the task and completed the exposure phase. The overview consisted of giving participants instructions on the mechanics of the task in each phase and provided time for them to ask the experimenter any questions. The exposure phase consisted of watching a video of first person movement along the perimeter of the city (**Figure 1A**). The video stopped for 5 s at each of the target landmarks and the experimenter pointed to the landmark on the screen and verbally confirmed that the participant had seen it before proceeding. The video stopped at the same point it had started, which was a randomly selected point along the perimeter and was consistent across participants. The exposure phase was designed to give the participants a sense of scale of the city and a baseline knowledge of the target landmark identities and locations.

Once inside the scanner, participants completed the encoding phase. This phase consisted of a sequence of fixation, reinstatement, and navigation blocks (**Figure 1C**). It began by placing participants at a random starting point in the city (randomized once and held consistent across participants), showing them an image of one of the target landmarks, and asking them to rate on a scale of 1–4 their confidence in knowing the location of the landmark (1: not at all, 4: very well). This is termed the reinstatement block. Afterward, the participants were instructed to find the cued landmark as quick as possible. This is termed the navigation block. Once located, the participants walked into the front of the building which prompted the next rest/reinstatement/navigation block sequence. We also included a helper arrow that was initiated once participants had taken more than 90 s to locate the landmark. The helper arrow always pointed in the cardinal direction of the cued landmark, requiring the participants to still make decisions on how best to navigate to it. The helper arrow was included based on results from an initial pilot study that showed trials where participants took longer than 90 s frequently resulted in getting lost. Optimal path time between each landmark pair was calculated by taking the quickest possible path between landmarks using available shortcuts (mean path time for optimal routes = 24.85 s, *SD* = 7.15; mean number of turns = 5.9, *SD* = 2.33). As with the starting point, the order of starting-goal landmark pairs was randomized once and held consistent across participants. The encoding phase lasted for 23 min and had an upper limit of 21 possible trials consisting of all pairwise combinations of landmarks in both directions, as well as the initial starting trial. At the end of the 23 min, scanning stopped irrespective if they had completed all the trials or not.

After the encoding phase, participants completed the simulation phase (**Figure 1D**). All participants conducted two practice trials prior to entering the scanner to ensure they properly understood the task instructions and provide them with the opportunity to ask the experimenter questions. Participants were instructed that the simulation portion would begin with the word ‘Simulation’ on the center of the computer screen. Afterward, they would be shown two images of the five target landmarks – one on the left and one on the right. Once the landmarks disappeared from the screen, they were instructed to mentally simulate in as much detail as possible moving through the city from the landmark on the left to the one on the right. The experimenter emphasized that it was important to mentally immerse themselves in the city and to take as much time as they needed to properly navigate the route. Participants were instructed to mentally navigate the quickest route between landmarks rather than trying to specifically recall the route they had previously taken.

Of critical importance here, participants were not instructed to simply try and remember their initial route between landmarks in the encoding phase. The reason for this is twofold. First, routes between landmarks in the encoding phase occurred with different levels of environmental familiarity due to their place in the trial order. As such, simple replay of past experiences during simulations are not in all cases representative of the fastest possible routes between two landmarks. Second, the interest here is in predictive simulations rather than memory replay. Simulations allow participants to incorporate spatial information they’ve learned throughout the experiment rather than trying to recall specific instances of an episode.

Inside the scanner, each trial of the simulation phase began with a fixation period for 12 s in which a white fixation cross was placed on top of a black background. Next, the word ‘Simulation’ displayed on the screen for 2 s. Following that, images of the starting and target landmark appeared for 3 s. Immediately afterward, the screen turned to black and the participants began to mentally simulate the route. The simulation period lasted for 15 s. The length of this block was determined using a larger behavioral study that recorded precise simulation times using the same experimental paradigm (Arnold et al., 2016). In that study, we observed that the average simulation period was approximately 15 s (*SD* = 11.21). Importantly, we also observed no statistical differences in simulation length comparing high (mean simulation time = 12.11 s, *SD* = 9.55) and low (mean simulation time = 14.5 s, *SD* = 14.03) vividness simulations [*t*(127) = 0.96, *p* = 0.34], nor were there when comparing high (mean simulation time = 12.94 s, *SD* = 10.72) and low (mean simulation time = 15.04 s, *SD* = 13.44) spatial coherence simulations [*t*(98) = 0.8, *p* = 0.42]. This suggests that potential confounds due to participants engaging in other forms of cognition after completing a simulation, but before the block has ended, are consistent across all levels of the analyses and therefore will have a minimal impact on the data. After the simulation, participants complete a 14 item post-simulation questionnaire (PostSQ). **Table 4** outlines the wording for each question/response and how they were grouped into different factors for analysis. The PostSQ included items modified from the Memory Characteristics Questionnaire (Johnson et al., 1988), as well as novel items, and was intended to probe qualitative aspects of the simulation experience. This included questions about spatial and temporal coherence, vividness, fractionation, confidence in knowing the starting/target locations, and perceived accuracy of their memory for the route. Each item was rated on a scale of 1–4. Immediately following the questionnaire, participants were placed within the virtual city facing the starting landmark and navigated to the target landmark as quick as possible. Once there, a post navigation questionnaire (PostNQ) was displayed where they rated two items on a scale of 1–4 assessing how well they simulated the route and how well the simulation matched their navigation experience. In total, 10 routes were included in the simulation phase. The starting-destination landmark pairs were pseudo-randomly selected such that each of the five landmarks were included as a starting point and destination only once.

### Functional MRI Data Acquisition

All MRI data were collected using a 3T GE Discovery MR750w scanner with a 32-channel head coil. A single shot EPI sequence was used, consisting of 38 interleaved T2*-weighted slices per volume (flip angle: 77°, TR: 2000 ms, TE: 30 ms, 3.6 isotropic voxel size, 64×64 matrix size). The first five volumes were discarded to allow for T1 equilibrium. Additionally, a T1-weighted three dimensional FSPGR anatomical image using 1 mm isotropic voxels was collected to assist with normalization of the EPI data.

### Functional MRI Preprocessing

All fMRI data were preprocessed through Nipype (Gorgolewski et al., 2011) using FSL (version 5.0.9^2^) and Advanced Normalization Tools (ANTs^3^). Data were first realigned with MCFLIRT, smoothed using a 7 mm FWHM Gaussian filter, intensity normalized, and temporally filtered using a 90-s high-pass filter for the encoding phase data and a 60-s filter for the simulation phase data. Next, all data were denoized using MELODIC to remove non-hemodynamic components based on inspection of the time course and power spectrum for each component. Anatomical and EPI data were then normalized into MNI152 space and resliced into 2 mm voxel space using ANTs by first computing a transformation matrix for registering each participant’s anatomical image to the MNI152 2 mm template, and then applying a linear transform of each EPI volume using the computed matrix. To further minimize non-BOLD signal from the data and to normalize the distribution of correlation values between ROIs, anatomical images were segmented into gray matter, white matter, and cerebral spinal fluid (CSF) estimates. These tissue classes were then used to apply the CompCor noise correction method (Behzadi et al., 2007) by regressing out principle components obtained from each participant’s white matter and CSF estimates from signal located in gray matter.

### Node Definition

Three hundred and thirty-three regions of interest (ROIs) spanning across the entire cortex were obtained from resting-state functional connectivity boundary mapping conducted by Gordon E.M. et al. (2014). We additionally included subject-specific ROIs for the left and right HC for a total of 335 ROIs in the analyses. These hippocampal ROIs were obtained through segmenting subcortical regions in native space for each participant’s anatomical scan using FIRST (Patenaude et al., 2011), and then applying the transformation matrix calculated from ANTs to resample and register each ROI to MNI152 2 mm space.

### Graph Construction

Correlation matrices for all network analyses were calculated using the CONN toolbox inside SPM12. A series of general linear models (GLMs) were constructed using the encoding phase data by binning reinstatement and navigation periods at the trial level based on the confidence rating for each trial. That is, for each participant we binned their reinstatement and navigation blocks into four levels by how confident the participant was in knowing the target landmark location (1: not at all, 4: very well). For the simulation phase data, simulation period blocks were binned into four levels based on participant ratings for (i) simulation vividness (a composite measure of six questions; see **Table 4**), and (ii) the spatial coherence of featural information during a mental simulation (1: vague, 4: clear/distinct). Each GLM was then convolved with the canonical hemodynamic response function in SPM12 and used to calculate 335×335 correlation matrices using Fisher transformed *r*-values.

### Graph Analyses

Calculation of all graph metrics was done using the brain connectivity toolbox for Python (version 0.4^4^). Briefly, a graph *G* (*N,E*) is characterized as a set of *N* nodes (here, 335 ROIs) and *E* edges (here, Fisher transformed *r*-values) representing the relationship between time varying data in any pairwise combination of nodes. Graphs are represented as a correlation matrix *C_ij_* where *i, j* is defined by the number of nodes being analyzed. We analyzed a number of graph metrics for both global and local networks. A global network is composed of the entire set of nodes that share at least one direct connection with another node, while a local network is defined as a subset of nodes that share some form of connection with a specific node. Both left and right hippocampal nodes were used to define local networks in the present study.

Each graph metric was calculated across a range of density levels by thresholding each correlation matrix *C_ij_* based on a series of cost values (*k*). Cost thresholds are applied to isolate a fixed percentage of edges (i.e., connections) between nodes in a graph. For the present study, we investigated each graph metric across a *k* value range of 0.1–0.25 at 0.05 increments representing the top 10–25% edges in each graph, a similar range used to identify developmental (Khundrakpam et al., 2013) and clinical (Bassett et al., 2008) changes in network topology. Each thresholded correlation matrix was then binarized by setting all supra-threshold edges to 1 and all sub-threshold edges to 0 to produce an adjacency matrix used for calculating different graph metrics.

### Global Network Metrics

We calculated three metrics to investigate reorganization of global network topology: modularity, global efficiency, and global flow. Modularity was calculated using a spectral community detection algorithm developed by Newman (2006). Modules are defined as a subset of nodes in a graph *G (N,E)* such that nodes within a module are more densely connected than between modules. Partitioning of a graph into modules is done by maximizing the modularity index *Q* by iterating over possible sub-divisions of a network. *Q* is obtained by first calculating a modularity matrix *B_ij_* using the formula:

$$\begin{array}{c} B_{ij}\;=\;A_{ij}-\frac{k_{i}k_{j}}{2m} \end{array}$$

where *A_ij_* is the observed number of edges between node *i* and *j*, and $\frac{k_{i}k_{j}}{2m}$ is the expected number of edges in a random graph where *k_i_* and *k_j_* are the degrees of each node and $m=\frac{1}{2}\sum_{i}k_{i}$ is the total number of edges in the graph. The modularity matrix is then used to find the most positive eigenvalue and corresponding eigenvector. Next, the graph is subdivided into two parts based on the signs of the elements in the vector and repeated for each of the parts using a general modularity matrix defined as:

$$\begin{array}{c} B_{ij}^{\left(g\right)}=B_{ij}-\delta_{ij}\sum_{k\in g}B_{ik} \end{array}$$

where $B_{ij}^{\left(g\right)}$ is the matrix indexed by *i,j* within group *g.* See Newman (2006) for a complete overview of the algorithm and a full description of the parameters used to optimize *Q*.

Global efficiency is the inverse characteristic path length in a graph ([.e., the average shortest path length between any two pairs of nodes (Watts and Strogatz, 1998)]. In functional brain networks, it represents the minimum number of statistical associations required to link any two brain regions and is indicative of the integrative and communicative capacity of a network to share information (Sporns et al., 2007; Bullmore and Sporns, 2009; van den Heuvel and Hulshoff Pol, 2010). Its inclusion in the present analysis is to provide a metric of global integration across all nodes in the network. Global flow is the average flow coefficient (Honey et al., 2007) across all nodes in a graph and represents the degree to which, on average, nodes act as hubs within local neighborhoods. Its inclusion here is quantify integration at a local scale.

### Node Centrality Metrics for Left and Right HC

To investigate the role of the left and right hippocampal nodes within global and local networks, we calculated four commonly used metrics: betweenness centrality, flow coefficient, the participation coefficient, and local efficiency. Betweenness centrality is the number of shortest paths in a network that pass through a specific node and indicates the importance of a node to global processing in a network. It is calculated with the formula:

$$\begin{array}{c} b_{i}=\frac{1}{\left(n-1)(n-2\right)}\sum_{{}_{\begin{array}{l} \;\;\;\;\;\;\;h,j\in N\\ h\neq i,h\neq j,i\neq j \end{array}}}\frac{\rho_{h,j}^{\left(i\right)}}{\rho_{h,j}} \end{array}$$

where ρ_h,j_ is the number of shortest paths in a graph that pass between *h* and *j*, and $\rho_{h,j}^{\left(i\right)}$ represents the number of shortest paths between *h* and *j* that pass through node *i*.

The flow coefficient is a measure of local efficiency (Honey et al., 2007) that quantifies the fraction of all paths with a length of two divided by the total possible number of paths with length two that traverse a node. It is calculated as:

$$\begin{array}{c} FC=\frac{p_{o}}{p_{p}} \end{array}$$

where *p*_0_ is the number of actual paths with a length of two and *p_p_* is the number of possible paths with a length of two.

The participation coefficient quantifies the amount of inter-module connections for a node such that nodes with a high participation coefficient act as connector hubs in a modular network by integrating processing across different communities. The participation coefficient is calculated as:

$$\begin{array}{c} y^{i}=1-\sum_{m\in M}{\left(\frac{k_{i}(m)}{k_{i}}\right)}^{2} \end{array}$$

where *M* is the set of modules identified using a community detection algorithm, and *k_i_*(*m*) is the number of edges between node *i* and all nodes in module *m*.

Local efficiency is similar to global efficiency but is calculated using a subset of nodes that share a direct connection with a particular node. As such, in functional networks it may be thought of as quantifying communication capacity of a network centered on a particular brain region. It is defined by Latora and Marchiori (2001) as the efficiency of a subgraph *G_i_* composed centered on the *i*th node, where the subgraph is composed solely of nodes that are immediate neighbors of *i*. It is calculated using the formula:

$$\begin{array}{c} E_{loc}(i)=\frac{1}{N_{G_{i}}(N_{G_{i}}-1)}\sum_{j,h\in G_{i}}\frac{1}{l_{jh}} \end{array}$$

where *l_jh_* is the shortest path length between nodes *j* and *h*, and *N_Gii_* is the number of nodes in the subgraph *G_i_*.

### gPPI Data Analysis

To address the *feature reinstatement* hypothesis, fMRI data were analyzed at the bivariate level using generalized psychophysical interaction (gPPI) models and at the multivariate level using graph theoretical measures. Generalized PPI models allow for the assessment of context-specific changes in functional connectivity between a seed region and sets of voxels across the brain (McLaren et al., 2012). Models are constructed by taking the interaction between the time course of the seed region and a GLM describing a task context, and searching for sets of voxels with a time course that correlates to the interaction model. Here, we use right and left hippocampal seeds defined using subject-specific segmentations generated using the FIRST algorithm in FSL to investigate context-specific changes in functional connectivity between simulation and navigation periods, as well as between simulation periods with different levels of reported vividness and spatial coherence. All gPPI analyses were conducted as whole brain analyses and used the standard corrections for multiple comparisons with a voxel height threshold of *p* < 0.001 and a cluster threshold of *p_FWE_* < 0.05.

## Results

### Behavioral Performance

The experiment began with an exposure phase in which participants viewed a video of passive first person movement along the perimeter of the city (**Figure 1A**). Following that, participants completed the encoding phase in the scanner (**Figure 1C**). Each trial began by cueing a target landmark and asking participants to rate their confidence in knowing its location within the city (termed the recall block), after which they were asked to navigate to the cued landmark as quickly as possible (termed the navigation block). All analyses in this manuscript that use data from the encoding phase were conducted on the navigation blocks. A total of 14 participants completed on average 13.14 trials (*SD* = 4.91) of 21 possible trials during the encoding phase. Mean path time for the navigation block was 82.7 s (*SD* = 69.24) and the average length of time during the recall block was 2.85 s (*SD* = 2.09). There was a total of 69 trials in the low confidence bin and 50 in the high confidence bin. Path number (i.e., whether a path occurred at the beginning or end of the encoding phase) and confidence rating were significantly correlated [*r*(117) = 0.55, *p* < 0.001], indicating that low confidence trials occurred early in the encoding phase and confidence ratings increased with exposure to the environment. Confidence ratings were negatively correlated with observed path time [*r*(117) = -0.32, *p* < 0.001] and path efficiency [*r*(117) = -0.31, *p* < 0.001], indicating that higher confidence in knowing landmark locations was associated with more efficient navigation.

Following the encoding phase, participants performed the simulation phase. Here, participants were cued with a starting and destination landmark from the encoding phase and given 15 s to mentally simulate a route between them in as much detail as possible. After the simulation, they were placed in front of the starting landmark within the virtual city and asked to navigate to the target landmark as fast as possible. Participants completed an average of 7.79 trials (*SD* = 2.26) out of 10 possible trials and spent an average of 1112.37 s (approximately 18 min and 30 s; *SD* = 84.2 s) completing the simulation phase. Mean path time was 60.8 s (*SD* = 53.98). Simulation vividness (mean rating = 2.96, *SD* = 0.75) and spatial coherence (mean rating = 2.9, *SD* = 0.99) were significantly correlated [*r*(102) = 0.52, *p* < 0.001], suggesting that highly vivid mental simulations are also spatially ordered. We also investigated whether simulation vividness and spatial coherence correlated to observed path time for each of the subsequent routes. Here, we found a statistically significant negative correlation [*r*(102) = -0.41, *p* < 0.001] between simulation vividness and observed path time, as well as one between simulation spatial coherence and observed path time [*r*(102) = -0.2, *p* = 0.044]. This demonstrates that aspects of feature reinstatement are relating to behavioral performance on the task, where more vivid and spatially coherent simulations relating to quicker subsequent path times. We have outlined and discussed similar findings with a larger behavioral dataset more widely in a previous study (Arnold et al., 2016), where we suggest a model in which more effective feature integration relates to quicker simulation times and subsequently more efficient wayfinding. Importantly, 44 of the 102 (40%) routes completed in the simulation phase were not navigated during the encoding phase, and there was a non-significant correlation for path time between identical routes in the encoding and simulation phase [*r*(58) = 0.18, *p* = 0.14]. Together, these findings suggest that participants were not simply replaying memories from the routes in the encoding phase during mental simulations but instead simulating novel routes (Arnold et al., 2016).

### Global Network Reorganization During Encoding

Our *global integration* hypothesis predicts that functional networks across the brain will demonstrate dynamic reorganization as mnemonic representations are encoded, increasing in modularity and decreasing in the amount integration as encoding occurs. Briefly, graph theoretical measures are calculated primarily at three levels: (1) across a global network, where the number of nodes and edges being analyzed is the total set of nodes and edges in a network; (2) across local networks, which are subsets of nodes and edges within a global network that share some specific criteria (e.g., all nodes in a local network have a direct connection with a certain node); or (3) on individual nodes and the direct connections between that node and others in a global or local network. To investigate this hypothesis, we calculated three graph metrics at the global network level that are proxies for the degree of integration occurring at different stages of the encoding phase. These metrics were calculated first by binning trial-level navigation blocks (mean path time = 82.7 s) based on the confidence rating for knowing the target landmark location and compared them across a range of density thresholds. Confidence ratings during memory retrieval has been shown to act as an effective proxy for the engagement of memory-selective neurons in the HC that are involved in indexing mnemonic features within declarative memory systems (Eichenbaum et al., 2007; Rutishauser et al., 2015). Here, they are used to infer encoding demands. We reasoned that low confidence judgments indicate higher encoding demands as the participants need to encode more environmental information into their representations of the city, whereas high confidence judgments are the result of feature rich representations. Importantly, we included a choice during confidence ratings (confidence rating = 1; see “Materials and Methods”) for when participants were unsure of the target landmark location, in attempts to remove trials from the low confidence bin where the participant was simply guessing or did not attempt to retrieve the landmark location. In this context, we believe that the low confidence level (i.e., confidence level = 2) analyzed here is inclusive of only trials in which the participants had some sense of where the landmark may be located, but did not have a detailed memory of how to navigate there and therefore had higher demands on encoding spatial information.

**FIGURE 2:**
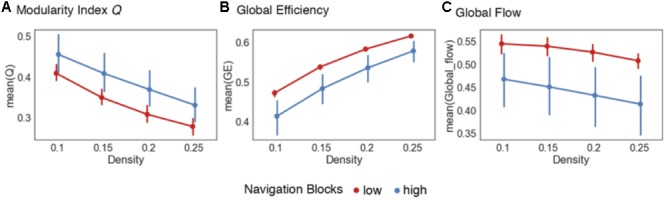
Global network reconfiguration during representation encoding. Graph metrics evaluating the *global integration* hypothesis. **(A)** Increases in the modularity index Q were observed across the whole 335 ROI network during high confidence navigation blocks across all the density thresholds (upper 10–25% of connections in the network). **(B)** Similarly, there were increased global efficiency values across the entire density range during the low confidence trials during navigation blocks. **(C)** Low confidence trials were also associated with increased values of global flow for each density threshold. Error bars represent 95% confidence intervals.

The first graph metric analyzed was the modularity index *Q* (Newman, 2006), a measure of the degree of modularity observed in a network. Briefly, higher values of *Q* indicate that a global network has a more robust modular structure (see “Materials and Methods” section on global network metrics for details on the algorithm used to compute *Q*). Modularity is theorized to provide a mechanism for adaptability in the brain (Ghosh et al., 2008; Meunier et al., 2010; Werner, 2010), with low levels of modularity relating to a higher capacity to integrate information across a global network. Previous research has associated changes in modularity with motor learning tasks (Bassett et al., 2011) and working memory paradigms such as the n-back task (Stanley et al., 2014; Cohen and D’Esposito, 2016). Our *global integration* hypothesis uses this perspective to predict that there would be an increased modular structure within the brain as mnemonic representations of the virtual city are encoded. We found support for this hypothesis with the modularity index *Q*. For the navigation blocks, there was a statistically significant difference using paired-samples *t*-tests the majority of density thresholds (see **Table 1** for complete list of statistics). We also computed a summary metric by collapsing across all density thresholds and comparing differences of *Q*. There were also statistically significant differences in *Q* using this summary metric, [*t*(55) = -4.87, *p* < 0.001]. **Figure 2A** summarizes these results.

**Table 1:** Statistical results across density thresholds for the global integration hypothesis.

Hypothesis	Graph metric	Density *(k)*	*t*-statistic	*p*-value
*Global integration*				
	*Q*	0.1	–1.85	0.087
		0.15	–2.57	0.023
		0.2	–2.63	0.021
		0.25	–2.5	0.026
	*Global efficiency*	0.1	2.5	0.027
		0.15	2.64	0.02
		0.2	2.66	0.02
		0.25	2.65	0.02
	*Global flow*	0.1	2.32	0.38
		0.15	2.54	0.024
		0.2	2.69	0.019
		0.25	2.72	0.018

Next, to complement the modularity analysis, we computed the global efficiency values for each graph across the different density thresholds. Global efficiency is calculated at the global network level and represents the integrative and communication capacity of a network by indicating, on average, how interconnected nodes in a network are (Sporns et al., 2007; Bullmore and Sporns, 2009; van den Heuvel and Hulshoff Pol, 2010). More generally, high global efficiency networks are characterized by short path lengths (i.e., the number of edges needed to connect any two nodes in a network), indicating high levels of information integration, and has been linked to the capacity to recall spatial features from memory (Arnold et al., 2014b). As with modularity, our prediction here was that higher encoding demands on low confidence trails compared to high confidence ones would require more integration of information processed across the brain and therefore be related to higher levels of global efficiency. We found support for this prediction across all density thresholds (**Figure 2B**). There were statistically significant differences across all density thresholds (**Table 1**) and for the summary metric [*t*(55) = 5.23, *p* < 0.001].

Thus far the data suggest that as encoding demands decrease, brain networks reorganize into a more modular state coupled with a reduction in global integration. Another important aspect of information flow in networks is based on the topological structure of local networks (i.e., neighborhoods). Local networks in the brain are subsets of nodes (i.e., parcellated brain regions) that share some characteristic, such as a statistically significant functional correlation with a certain node, and do not incorporate information about the spatial distribution of nodes. That is, local networks can consist of spatially distant regions of the brain that have similar functional activations in response to a task. In the context of encoding mnemonic representations, it is plausible to suggest that higher encoding demands are also associated with increased need for processing within local networks early on. More specifically, as features of an environment are encoded into a representation, there may be a higher demand placed on not only integrating between, but also processing within, task-relevant systems such as the visual, somatosensory, and attentional subnetworks. To quantify and compare this, we calculated the global flow coefficient (Honey et al., 2007). This metric is the average flow coefficient of the complete set of nodes within a global network. The flow coefficient (Honey et al., 2007) represents how efficiently information flows between neighboring nodes and is therefore representative of integration within local networks. As such, the global flow coefficient represents the amount of integration occurring within the complete set of local neighborhoods in a global network rather than one neighborhood in particular. Here, the prediction was that higher values of global flow (and therefore more local integration) would be associated with the increased encoding demands of low confidence trials. As with modularity and global efficiency, we found support for our hypothesis (**Figure 2C**). There were statistically significant differences for all density thresholds (**Table 1**) and for the summary metric [*t*(55) = 5.28, *p* < 0.001].

### Hippocampal Centrality During Encoding

**FIGURE 3:**
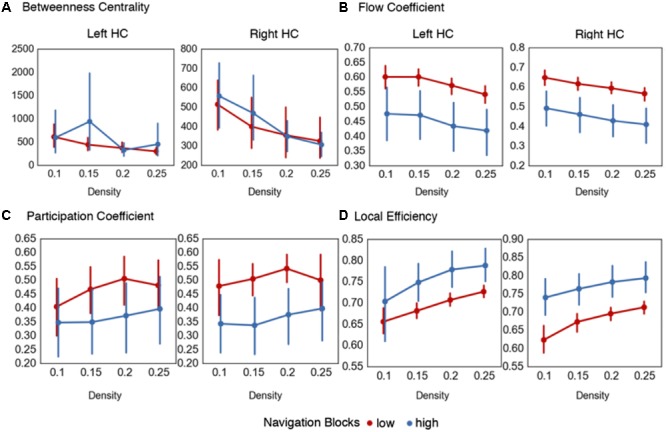
Centrality measures for hippocampal nodes during representation encoding. Graph metrics evaluating the *node flexibility* and *state transition* hypotheses. **(A)** No differences were observed across the density thresholds for the betweenness centrality of the left and right HC during navigation blocks. The summary statistic (betweenness centrality values averaged across density thresholds) was significantly increased during high confidence trials for the left HC during memory reinstatement. **(B)** Increased values of the flow coefficient were observed for both the left and right HC in low confidence trials during navigation blocks. **(C)** Across the majority of density thresholds (0.15–0.25) there were increased participation coefficient values during low confidence trials for the right HC during navigation blocks, but only for the summary statistic in the left HC. **(D)** Across all density thresholds in the right HC and for the majority (0.15–0.25) for the left HC, there were increased local efficiency values for the hippocampal sub-network during high confidence navigation blocks. Error bars in all graphs represent 95% confidence intervals.

The *node flexibility* hypothesis predicts that critical convergence zones such as the HC dynamically change functional interactions with other brain regions to alter the degree to which environmental information is integrated into a neural index. To test this, we investigated how the HC acts as a network hub at the global, inter-module, and local network level, and whether change in these measures relate to the reorganization of global brain networks while representations are formed. This was done by calculating four node-based metrics that quantify different aspects of hubness in a network using the navigation blocks (mean path time = 82.7 s) from the encoding phase that were binned by confidence rating, similar to the global graph metrics. The first metric was betweenness centrality, a common measure of global network centrality that quantifies the number of shortest paths between nodes that pass through a given node. This metric is calculated at the global network level, and indicates the importance of a node (i.e., here, the HC) to information flow in a global network by serving to connect any two nodes in a network. **Table 2** and **Figure 3A** summarizes these results. Here, we found no statistically significant differences at the different density thresholds during navigation blocks for the right [summary statistic: *t*(55) = -0.38, *p* = 0.7], or left HC [summary statistic: *t*(55) = -1.12, *p* = 0.27]. The lack of statistical differences here suggests that when considering the brain as a single, global network, there are no differences in HC centrality. However, as demonstrated in the previous section, the brain displays a modular structure during this task and therefore changes in HC centrality may only occur within and between modules.

**Table 2:** Statistical results across density thresholds for the node flexibility hypothesis.

Hypothesis	Graph metric	Hemisphere	Density *(k)*	*t*-statistic	*p*-value
*Node flexibility*					
	*Betweenness centrality*	Right	0.1	0.32	0.75
			0.15	0.5	0.63
			0.2	0.07	0.95
			0.25	0.25	0.81
		Left	0.1	0.07	0.95
			0.15	1.13	0.28
			0.2	0.66	0.52
			0.25	0.85	0.41
	*Flow coefficient*	Right	0.1	3.08	0.009
			0.15	3.15	0.008
			0.2	3.26	0.006
			0.25	3.07	0.009
		Left	0.1	2.44	0.03
			0.15	3.05	0.009
			0.2	3.42	0.005
			0.25	3.09	0.009
	*Participation coefficient*	Right	0.1	1.97	0.07
			0.15	2.79	0.015
			0.2	2.92	0.01
			0.25	1.45	0.17
		Left	0.1	0.77	0.46
			0.15	1.65	0.12
			0.2	1.9	0.08
			0.25	1.31	0.21

To expand on this, we calculated the flow coefficient (Honey et al., 2007), a graph theoretical measure that quantifies the centrality of a node within a local network. High values of the flow coefficient indicate that a particular node (here, the HC) is involved in connecting any other two nodes in a local network, and therefore acts as hub for information flow. **Table 2** and **Figure 3B** shows the results. We found statistically significant differences across all density thresholds for the right [summary statistic: *t*(55) = 6.45, *p* < 0.001] and left HC [summary statistic: *t*(55) = 6.45, *p* < 0.001].

The two analyses so far suggest that the HC displays increased centrality during navigation when encoding demands are high within a local neighborhood composed of regions that share a functional connection with the HC, but not when treating the brain as a global network. Given that there were observed shifts in modularity associated with encoding demands, it is plausible that the HC is acting as a connector hub integrating information across these modules rather than as a hub across a singular whole brain network. To investigate this, we calculated the participation coefficient (Guimerà and Amaral, 2005). The participation coefficient quantifies the amount of inter-module connections of a node compared to the amount of intra-module connections, and is representative of the degree to which a node participates in and integrates across different subnetworks. These results are summarized in **Table 2** and **Figure 3C**. The participation coefficient increased when encoding demands were high across most of the density thresholds and the summary statistic for the right HC [*t*(55) = 4.54, *p* < 0.001], but only with the summary statistic for the left [*t*(55) = 2.85, *p* = 0.006].

### Global to Local State Transitions

Lastly, the local efficiency for hippocampal subnetworks was calculated to test the *state transition* hypothesis. Local efficiency (Latora and Marchiori, 2001) is conceptually similar to global efficiency, in that it quantifies the degree to which any two nodes in a network are connected, but is calculated on a local network defined as nodes sharing a direct connection with the HC. High values of local efficiency represent an increased capacity to integrate information across nodes in a local network (Rubinov and Sporns, 2010). As outlined previously, the prediction was that as participants form representations during encoding, there should be reconfiguration of network topology during navigation from a state supportive of global integration to one based on local network processing. Again, we used the navigation blocks from the encoding phase (mean path time = 82.7 s) binned by confidence rating. We found support for this prediction (**Table 3** and **Figure 3D**) with increased local efficiency in high confidence navigation blocks for the right [summary statistic: *t*(55) = -6.76, *p* < 0.001] and across the majority of density thresholds for the left HC [summary statistic: *t*(55) = -4.21, *p* < 0.001].

**Table 3:** Statistical results across density thresholds for the state transition hypothesis.

Hypothesis	Graph metric	Hemisphere	Density (*k*)	*t*-statistic	*p*-value
*State transition*					
	*Local efficiency*	Right	0.1	–3.76	0.002
			0.15	–3.15	0.008
			0.2	–3.25	0.006
			0.25	–3.08	0.009
		Left	0.1	–1.04	0.32
			0.15	–2.58	0.022
			0.2	–3.36	0.005
			0.25	–3.04	0.01

### Hippocampal–Cortical Interactions During Prospective Mental Simulation

Retrieval and integration of environmental features from memory into the spatiotemporal context for prospective mental simulation is believed to operate through the reinstatement of regional activity in sensory and associative areas of the cortex, coordinated primarily through pattern completion and separation mechanisms in the HC (Norman and O’Reilly, 2003; Stokes et al., 2014; Horner et al., 2015). Based on this perspective, we formulated the *feature reinstatement* hypothesis and predicted that there would be increased functional coupling between the HC and areas of visual cortex during simulations with high visual and spatial fidelity. To test this, we binned the 15-s simulation blocks based on how participants rated the simulation vividness and spatial coherence in the post-simulation questionnaire (PS-Q; **Table 4**). This binning strategy was done on the simulation phase data that was collected at the completion of the encoding phase. The binned simulation blocks were then used to construct gPPI models (McLaren et al., 2012) by multiplying the time course of BOLD signal in the left and right HC with GLMs denoting trials with low (PS-Q rating value of 1) and high (PS-Q rating value of 4) vividness and spatial coherence. All gPPI analyses were conducted across the whole brain and were data driven (Biswal et al., 2010), as opposed to using *a prior* ROIs, due to the novelty of research into the neural mechanisms supporting mental simulations which putatively involve interactions across a wide set of brain regions (Brown et al., 2016).

**Table 4:** Post simulation questionnaire (PS-Q) items listed by feature integration process.

*Post-simulation Questionnaire*
Vividness
My memory for this route is (1: sketchy – 4: very detailed)
*My memory for this route is (1: entirely in color – 4: black and white)
My memory for this route involves visual detail (1: little or none – 4: a lot)
Overall vividness of this route is (1: vague – 4: very vivid)
My memory for this route is (1: dim – 4: sharp/clear)
When imagining this route, it was so vivid I felt I was actually navigating it (1: not at all – 4: a great deal)
Spatial Coherence
*The relative spatial arrangements of buildings along the route is (1: clear/distinct – 4: vague)
Temporal Coherence
The order of buildings along the route is (1: confusing – 4: comprehensible)
Fractionation
Simulating the route was like watching a movie in my mind’s eye (1: not at all – 4: very much)
The route was a collection of separate images (1: very much – 4: not at all)
Simulation Confidence
I have doubts about the accuracy of my memory for this route (1: a great deal – 4: no doubts)
Post Route Accuracy
My memory for this route matched my experience (1: not at all – 4: very well)
I was able to mentally simulate this route (1: not at all – 4: a lot)
Other
The route seems (1: long – 4: short)
*My memory of the starting location for this route is (1: clear/distinct – 4: vague)
My memory for the destination location for this route is (1: vague – 4: clear/distinct)

*Indicates response that was inverted prior to analysis.

**FIGURE 4:**
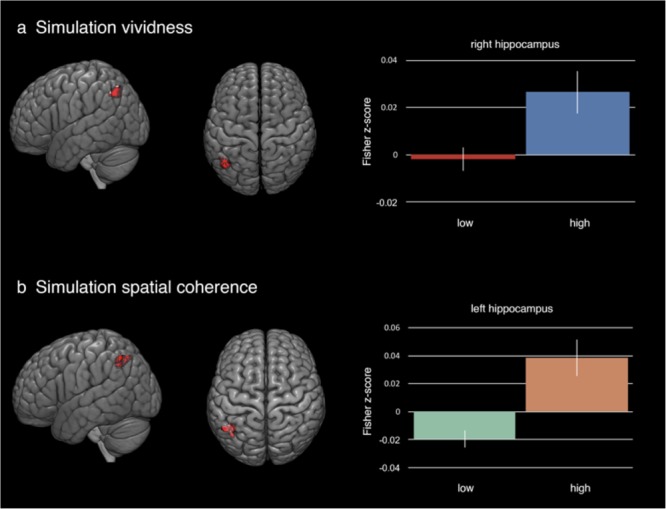
Results from general psychophysiological interaction analyses on mental simulations. **(a)** Simulations with high visual vividness were found to have increased functional connectivity between the right HC and a cluster located in the superior division of the left lateral occipital cortex. Graph on right shows Fisher transformed z-scores for the low vividness and high vividness simulation periods. **(b)** Similarly, increased functional connectivity was observed between the left HC and a cluster within both left angular gyrus and the superior division of the lateral occipital cortex. Fisher transformed z-scores for high and low spatial coherence simulations are on the right. **Table 5** lists regions and number of voxels per region for each cluster. Statistically significant clusters were identified using a voxel height threshold of *p* < 0.001 and a cluster threshold of pFWE < 0.05. Error bars represent standard error of the mean.

During highly vivid simulation blocks, we found evidence for increased functional connectivity between the right HC and the superior portion of the left lateral occipital cortex [*t*(13) = 4.81, *p* < 0.001; 128 voxels; peak MNI coordinates: -46, -64, 46] (**Table 5** and **Figure 4a**). For spatial coherence, high ratings for spatial coherence during simulations were associated with increased functional connectivity between the left HC and areas within the left angular gyrus and the superior division of the left lateral occipital cortex [*t*(13) = 4.34, *p* < 0.001; 118 voxels; peak MNI coordinates: -40, -56, 42] (**Table 5** and **Figure 4b**). There were no statistically significant increases in functional connectivity with the right or left HC in low vividness or spatial coherence simulation blocks. Considered together, these results support the *feature reinstatement* hypothesis that increased hippocampal–cortical functional coupling is associated with a high degree of simulation fidelity, putatively through more effective feature integration coordinated by the HC through selective functional coupling with areas of the brain associated with higher-order visual processing.

**Table 5:** List of brain regions showing increased functional connectivity related to simulation fidelity.

Analysis				Peak MNI coordinates (mm)				
	Source	Region	Hemisphere	*X*	*Y*	*Z*	Cluster size	Z-score change
Vividness	Right hippocampus	Superior lateral occipital cortex	Left	–46	–64	46	115	0.028
		Angular gyrus	Left				11	
Simulation	Left hippocampus	Angular gyrus	Left	–40	–56	42	62	0.058
		Superior lateral occipital cortex	Left				36	

*Table shows results for differences in functional connectivity between a source region and a multi-regional cluster. Anatomical regions for each cluster are listed by voxel size. All clusters were identified using a peak voxel threshold of *p* < 0.001 and a cluster correction threshold of *p_*FWF*_* < 0.05.*

### Network Topology and Simulation Fidelity

In the next analysis, we sought to extend the findings on changes in hippocampal–cortical functional coupling relating to simulation fidelity by testing the *feature integration* hypothesis. This hypothesis predicts that high simulation fidelity would be associated with hippocampal-based network states conducive to information integration. While bivariate techniques such as gPPI can elucidate the functional coupling between a seed region and a cluster of voxels sharing similar BOLD response patterns, multivariate techniques such as graph theory allow for the assessment of more complex patterns of information communication and integration by considering the functional interactions between more than two sets of regions in the brain (Sporns et al., 2007; Rubinov and Sporns, 2010; Bassett et al., 2012). Of importance here, graph theoretical measures allow for assessment of network dynamics in local neighborhoods (i.e., sub-networks characterized by shared patterns of functional interactions), and how a particular region (e.g., the HC) coordinates the information flow between multiple sets of regions. To assess how hippocampal network topology relates to simulation fidelity, we calculated node-based graph theoretical measures of betweenness centrality, flow coefficient, and local efficiency for the right and left hippocampal nodes. These measures quantify the centrality of the HC at the global and local network level, and assess the communication efficiency of local HC networks, respectively. Similar to the gPPI analysis, these measures were calculated by using the 15-s simulation blocks binned by either vividness or spatial coherence.

**FIGURE 5:**
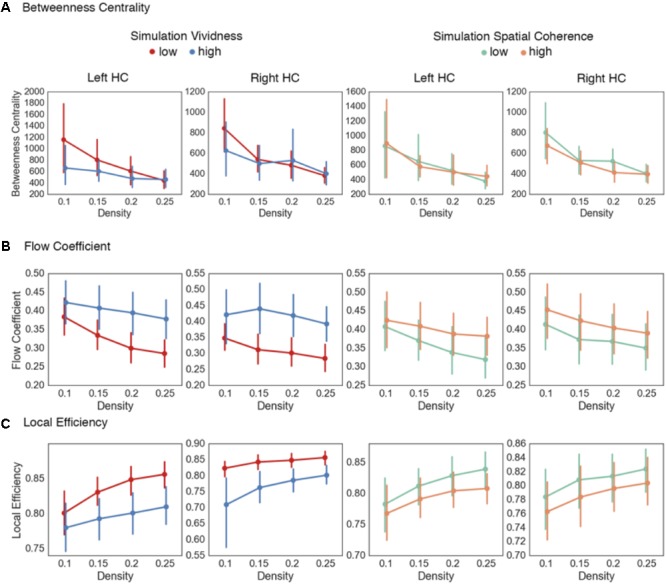
Results from hippocampal network analyses on mental simulations. **(A)** No statistically significant differences were found in the betweenness centrality of the left or right HC comparing either low and high vivid or spatially coherent mental simulations. **(B)** Increased flow coefficient values were found during highly vivid simulations in the right and left HC across higher density thresholds. **(C)** Decreased local efficiency values were found during highly vivid mental simulations in the right and across the higher density thresholds in the left HC. No statistical differences were observed between high and low spatial coherence mental simulations. Density thresholds represent the percentage of strongest connections in each network. Error bars represent 95% confidence intervals.

The first measure calculated was the betweenness centrality of the right and left HC. Comparing high and low vividness simulations (**Table 6** and **Figure 5A**), there were no statistically significant differences between betweenness centrality values across the density thresholds in the right [summary statistic: *t*(55) = 0.9, *p* = 0.37] or left HC [summary statistic: *t*(55) = 1.7, *p* = 0.09]. Similarly, comparing high and low spatial coherence simulations, there were no statistically significant differences across the density thresholds in the right [summary statistic: *t*(55) = 1.27, *p* = 0.21] or left HC [summary statistic: *t*(55) = -0.02, *p* = 0.99]. As with the confidence judgment analysis on the encoding phase data, there appears to be no differences in HC centrality when considering the brain as a single global network. However, given the modular structure of networks during the task, it is plausible that there are dynamic alterations in how the HC interacts between modules and within its local neighborhood.

**Table 6:** Statistical results across density thresholds for the global integration hypothesis.

Hypothesis	Graph metric	Fidelity metric	Hemisphere	Density *(k)*	*t*-statistic	*p*-value
*Feature integration*						
	*Betweenness centrality*	*Vividness*	Right	0.1	1.91	0.08
				0.15	0.43	0.67
				0.2	0.34	0.74
				0.25	–0.32	0.76
			Left	0.1	0.17	0.11
				0.15	1.02	0.33
				0.2	1.23	0.24
				0.25	–0.17	0.87
		*Spatial coherence*	Right	0.1	0.7	0.5
				0.15	0.37	0.71
				0.2	1.58	0.14
				0.25	0.11	0.91
			Left	0.1	–0.1	0.92
				0.15	0.39	0.71
				0.2	0.22	0.83
				0.25	1.02	0.33
	*Flow coefficien*	*Vividness*	Right	0.1	–1.31	0.21
				0.15	–3.21	0.007
				0.2	–3.45	0.004
	*t*			0.25	–3.34	0.005
			Left	0.1	–1.51	0.16
				0.15	–2.73	0.017
				0.2	–3.12	0.008
				0.25	–3.22	0.007
		*Spatial coherence*	Right	0.1	–0.77	0.46
				0.15	–0.99	0.34
				0.2	–0.74	0.47
				0.25	–0.88	0.4
			Left	0.1	–0.5	0.62
				0.15	–1.06	0.31
				0.2	–1.54	0.15
				0.25	–1.88	0.08
	*Local efficienc*	*Vividness*	Right	0.1	2.19	0.047
				0.15	3.76	0.002
				0.2	3.69	0.003
	*y*			0.25	3.42	0.005
			Left	0.1	1.42	0.18
				0.15	2.66	0.02
				0.2	3.07	0.009
				0.25	3.21	0.007
		*Spatial coherence*	Right	0.1	0.73	0.48
				0.15	0.86	0.41
				0.2	0.69	0.5
				0.25	0.87	0.4
			Left	0.1	0.74	0.47
				0.15	1.12	0.28
				0.2	1.48	0.16
				0.25	1.86	0.09

Next, we calculated the flow coefficient for the left and right hippocampal nodes (**Figure 5B**). In this analysis, the flow coefficient represents how central information flow vis-à-vis the HC is within its local network. Here, we found statistically significant increases in flow coefficients during high vividness simulations at the higher density thresholds (**Table 6** and **Figure 5B**) for the right [summary statistic: *t*(55) = -5.22, *p* < 0.001] and left HC [summary statistic: *t*(55) = -5.38, *p* < 0.001]. Comparing high and low spatial coherence trials, we found no statistical differences at the individual density thresholds (**Table 6** and **Figure 5B**). The summary statistic was statistically significant for the left [*t*(55) = -2.52, *p* = 0.01] but not the right HC [*t*(55) = -1.73, *p* = 0.09].

Thus far, the data show that there are no differences between the centrality of the HC at the global network level in mental simulations with high vs. low vividness and spatial coherence. However, there was evidence for increased hippocampal centrality within its local neighborhood during mental simulations with high vividness. Next, we sought to further evaluate information flow within hippocampal neighborhoods. As previously outlined, the HC is theorized to coordinate the selective reactivation of sensory and associative areas of the cortex using a neural index to reinstate environmental features from memory and integrate them into a representation used during mental simulation. A plausible prediction from this is that in trials with low hippocampal centrality (i.e., trials which tended to be correlated with low-vividness ratings), the coordination of feature reinstatement and integration is compensated by increases in functional interactions between other regions of the memory system supporting mental simulations (Fornito et al., 2012). To evaluate this, we calculated the local efficiency of hippocampal networks. Local efficiency represents the efficiency of information flow in a subnetwork composed only of immediate neighbors of a specific node (i.e., a neighborhood). Comparing simulations of high and low vividness (**Table 6** and **Figure 5C**), there were statistically significant decreases in local efficiency values for high vividness simulations in the right hippocampal neighborhood [summary statistic: *t*(55) = 5.17, *p* < 0.001], and across higher density thresholds in the left hippocampal neighborhood [summary statistic: *t*(55) = 5.22, *p* < 0.001]. We also investigated differences in local efficiency values of hippocampal networks between simulations with high and low spatial coherence. For the individual density thresholds, there were no statistically significant differences for the right or left hippocampal neighborhoods (**Table 6**). The summary statistic was significant for the left hippocampal neighborhood [*t*(55) = 2.6, *p* = 0.011], but not the right [*t*(55) = 1.6, *p* = 0.11]. Importantly, comparing the increased flow coefficient and decreased local efficiency of the right HC network during highly vivid simulations and the left HC for spatial coherence indicates that information flow vis-à-vis the HC, rather than increased information flow between all nodes in the HC network, is vital for highly vivid simulations.

## Discussion

Theoretical and computational models of memory function posit that mnemonic representations are generated by integrating sensory features processed across the neocortex into neural patterns within memory structures, and the retrieval of these representations involves reinstatement of feature-specific activity in the neocortex via pattern completion mechanisms in the HC (Marr, 1971; Damasio, 1989; McClelland et al., 1995; Nadel and Moscovitch, 1997; Norman and O’Reilly, 2003; Meyer and Damasio, 2009). While there is increasing evidence for these models during memory retrieval (Staresina et al., 2013; Watrous et al., 2013; Gordon A.M. et al., 2014; Schedlbauer et al., 2014; Horner et al., 2015; Backus et al., 2016), empirical evidence for how brain networks interact dynamically during encoding has been lacking. Here, using confidence ratings as a proxy of processing demands within memory networks (Rutishauser et al., 2015), we test a series of three hypotheses relating to dynamic network processes and how mnemonic representations are encoded and reinstated. The *global integration* hypothesis predicts that brain networks are in state of increased integration when encoding demands are highest. The *state transition* hypothesis predicts that as encoding progresses, brain networks transition from a state of global and inter-module integration into one emphasizing local processing within hippocampal networks. The third hypothesis, termed the *node flexibility* hypothesis, predicts that the convergence zones such as the HC flexibly alter functional connections with global and local networks, increasing in centrality as a global network and inter-module connector hub when encoding demands are high, and transitioning to local network processing once representations are formed. Collectively, the results from this study support these hypotheses and demonstrate for the first time that the topological structure of brain networks reconfigures from a state of global integration to localized processing based on the degree of integration of environmental information into a putative representation, and that the HC flexibly changes its role as an inter- and intra-module connector hub in response to these integrative demands. The subsequent use of mnemonic representations during prospection was also investigated using two hypotheses and shown to be associated with dynamic changes in network topology. During highly vivid and spatially coherent simulations, the HC was found to increase functional interactions with areas of left occipital cortex and angular gyrus that have previously been associated with object recognition, manipulation of mental imagery, and awareness of intended action sequences. Highly vivid simulations were also found to increase hippocampal centrality in local memory networks, indicating that the HC is critical for supporting multi-regional integration of visual information during prospection. Collectively, these results suggest that dynamic shifts in global and local network topologies, coordinated in part by changes to functional interactions with the HC, relate to the degree to which environmental information is encoded in a mnemonic representation.

Adaptability of complex networks is thought to operate in part through the dynamic formation and interaction of different network communities (Ghosh et al., 2008; Meunier et al., 2010; Werner, 2010; Bassett et al., 2011), allowing the network to optimize its output based on relevant environmental demands. Using 335 ROIs distributed across the brain, we found support for network adaptability during the encoding and retrieval of mnemonic representations. During navigation trials in which participants had low confidence in knowing the target landmark location, we observed lower values of the modularity index *Q* compared with trials in which they had high confidence in knowing the landmark location. This suggests that the brain displays an increasingly stable modular topology as the need to integrate environmental features lessens and can dynamically reconfigure its community organization based on changing task demands. This result was extended at the global and local network level, with low confidence navigation trials associated with increased values of global efficiency and global flow across the brain. This pattern of results supports the *global integration* hypothesis and provides empirical support at the network level for the long held but sparsely tested hypothesis that encoding features into mnemonic representations increases the integration of information processed in a distributed set of systems across the brain (Marr, 1971; Damasio, 1989; Squire and Zola-Morgan, 1991; McClelland et al., 1995; Nadel and Moscovitch, 1997; Meyer and Damasio, 2009). Building on the perspective of Bassett et al. (2011), as well as research on motor learning (Bassett et al., 2011) and working memory (Stanley et al., 2014; Cohen and D’Esposito, 2016), we suggest that adaptability in network topology underlies changes in how domain-specific information is integrated into holistic representations in a manner that allows the contents of a specific representation to become more stable over time. Further, decreases in global efficiency and increases in hippocampal community local efficiency indicate that as a representation is encoded, there is a decreased need to integrate across sensory and associative systems in the brain and an increased need to rapidly propagate information within the hippocampal sub-network. Although more research is needed, particularly in non-spatial memory paradigms, these findings provide a tentative experimental framework for understanding the neural basis of the dynamic formation of networked representations (Eichenbaum, 2000).

The HC has long been thought to be a primary convergence zone (Eichenbaum, 2000; Meyer and Damasio, 2009; Mišić et al., 2014; Backus et al., 2016), receiving multisynaptic inputs from both sensory cortices and associative systems in the perirhinal and parahippocampal cortex. This allows for conjunctive coding of high-level sensory and associative environmental features, such as spatial information to specific locations (O’Keefe and Nadel, 1978; Ekstrom et al., 2003) and the temporal sequence of places and events that form the basis of episodic memories (Eichenbaum, 2004, 2013; Davachi, 2006; MacDonald et al., 2011). Although the results of the current study are consistent with the role of the HC as a convergence zone, the current findings extend past results and support the *node flexibility* hypothesis by showing that the HC demonstrates flexibility during representational encoding by altering the degree to which it acts as a connector hub within local networks, as well as between network modules. On low confidence trials where encoding demands are highest, we observed increased values of the flow coefficient, indicating that the centrality of the HC within its local network is associated with the need to integrate sensory and associative information. Importantly, we also observed increased values of the participation coefficient on low confidence trials in the right HC, supporting its role as an inter-module hub, combining information processed within different modules across the brain into a putative mnemonic representation. Conversely, on high confidence trials, we found evidence for the *state transition* hypothesis local efficiency increased within a hippocampal sub-network. Considered together, these results suggest that the convergence of information into the HC is mediated in part by associative demands during the encoding of a representation, and operates dynamically by changing the functional interactions within and between network modules. As representations are formed, the centrality of the HC decreases while the efficiency of information flow within hippocampal sub-networks increases. This finding builds upon past work positing that the learned associations of a mnemonic representation are related to the topological composition of functional interactions between brain regions (Buchel et al., 1999; Eichenbaum, 2000), putatively through the reconfiguration of hippocampal interactions that initially allow sensory and associative information to be bound into a holistic representation that is subsequently coded by the functional interactions between components of a hippocampal based sub-network. Additionally, the dynamic nature of cognition during navigation (Spiers and Maguire, 2008; Ekstrom et al., 2017) may provide additional demands on network reconfiguration by requiring that the brain rapidly apply different cognitive operations that are critical to wayfinding. In the context of the present study, this suggest that the navigation blocks during the encoding phase contain instances of different cognitive processes, such as periodic reinstatement periods, that cumulate in observable navigation behavior. However, this is speculative and future research using time sensitive imaging methods such as multi-band MRI or magnetoencephalography may be able to further detail how different components of navigation behavior relate to alterations in network processing.

The reinstatement of mnemonic representations is not a binary process. Rather, recapitulation of task-oriented representations during prospection vary in how orderly and vivid encoded information appears subjectively. Variability in representation fidelity is theorized to be associated with how effectively environmental features from previous experiences can be recapitulated into a mnemonic representation underlying prospection (Arnold et al., 2016). Based on this and other outlined theoretical perspectives (Hassabis and Maguire, 2009; Schacter et al., 2012), we formulated the *feature reinstatement* hypothesis that the representational fidelity of a prospective mental simulation would require increased coordination between the HC and visual areas of the brain, as the neural codes of the spatial context in the HC putatively coordinates the recapitulation of environmental features needing to be integrated in a task oriented manner. The results from the current study provide support for this hypothesis by showing that highly vivid and spatially coherent simulations involve increased functional coupling between the HC and cortical areas associated with object representation and the manipulation of mental simulations. Comparing simulations with high and low vividness ratings using gPPI models, we observed increased functional connectivity during highly vivid simulations between the right HC and the superior division of the left lateral occipital cortex and the left angular gyrus. Similarly, we found increased functional connectivity during simulations with high ratings of spatial coherence between the left HC and the left angular gyrus and the superior division of the left lateral occipital cortex, similar to the area identified in the vividness analysis. Lateral occipital cortex has previously been implicated in representing high-level visual features of objects and how they are localized in spatial contexts (Kourtzi and Kanwisher, 2001; Xu and Chun, 2006; Silk et al., 2010). Additionally, this area has been found to uniquely increase activity during mental simulations that involve self-referential processes in non-present timeframes (i.e., past, future, and imagined) (Nyberg et al., 2010). Angular gyrus has more widespread functional roles, acting as a multi-modal hub integrating multisensory information to allow for the manipulation of mental representations (Seghier, 2013) and subjective awareness of intended action sequences and their consequences in spatial contexts (Farrer et al., 2008; Arnold et al., 2014b). Considered together, these functional interactions suggest that the neural codes in the HC representing the spatial context of a location interact with visual and associative areas of the cortex to reinstate and organize environmental features from memory. Critically, the degree of these functional interactions relate to how effectively environmental features can be reinstated, integrated, and manipulated during simulation of movement within a spatial context. This suggests that the neural codes underlying prospection involve changes in interactions between the hippocampus and other cortical regions, particularly those previously shown to integrate multisensory information and act as a representational buffer for high level spatial information, rather than resulting from neural processes located solely in the hippocampus.

Prospection is theorized to rely on functional interactions between a multi-regional network across the brain (Hassabis et al., 2007; Schacter et al., 2012; Brown et al., 2016). To characterize the topological structure of these networks and how the HC is involved in coordinating information flow between network components, we sought to complement the gPPI analysis by investigating changes in hippocampal network topology associated with representational fidelity. Here, we tested the *feature integration* hypothesis that predicts feature reinstatement and integration requires network states allowing more efficient communication (Arnold et al., 2014b), particularly with increased hippocampal involvement in coordinating information flow. We did not find support for this hypothesis at the global network level, with no statistical differences in betweenness centrality values for the HC when comparing mental simulations with high or low vividness or spatial coherence ratings. However, within hippocampal neighborhoods, the right HC had increased centrality as measured by the flow coefficient during mental simulations with high vividness ratings and the left HC with spatial coherence, albeit only the summary statistic. Additionally, we observed decreased local efficiency, a measure of information flow between any two nodes in a neighborhood, in simulations with high vividness ratings within the right HC neighborhood, and those with high spatial coherence within the left HC neighborhood. The presence of increased hippocampal centrality and decreased local efficiency within hippocampal neighborhoods suggests that coordination of information flow vis-à-vis the HC within memory networks during prospection is critical, facilitating the recapitulation and integration of spatial features from memory into a goal-oriented mnemonic representation. Conversely, in simulations with low visual fidelity, the decreased role of the HC in coordinating information flow appears to be compensated for by increased functional interactions between other regions in the memory network. This compensatory mechanism may allow for partial recapitulation of environmental features from memory, albeit at a lower visual resolution than simulations with increased hippocampal coordination (Rosenbaum et al., 2009; Yonelinas, 2013).

## Conclusion

The present study provides novel empirical support for critical predictions by theoretical models on how mnemonic representations are formed and subsequently used in a goal-oriented manner. We show that on low confidence trials, which we infer as having increased encoding demands, the topological structure of the brain is organized to facilitate global and local information flow. As representations are encoded, the HC flexibly changes its functional interactions across the brain, decreasing its role as connector hub within its local sub-network and across network modules, while the information flow within the hippocampal community increases in efficiency. The ability to subsequently use mnemonic representations for prospection was also related to dynamic changes in network topology. As predicted, both aspects of representational fidelity were related to increased functional coupling between the HC and visual and associative areas of the brain, putatively allowing for more effective feature integration during mental simulation. Highly vivid and spatially coherent simulations were also found to be associated with both increased hippocampal centrality and decreased local efficiency within a hippocampal sub-network, suggesting that the visual basis of a mental simulation requires coordination of information processing via the HC into high-resolution mnemonic representations (Rosenbaum et al., 2009; Yonelinas, 2013). This provides a tentative theoretical framework to understand the dynamic nature of representational encoding and retrieval, through assessing changes in topological structure across global and hippocampal based brain networks. Critically, this study also provides the first direct empirical evidence that the neural representations underlying prospection are generated and manipulated through hippocampal–cortical functional interactions rather than neural codes in the HC alone. Future research will be able to use this framework to understand how the pathology of cognitive and neurodegenerative disorders impacts the topological structure of global and local brain networks during memory encoding and retrieval, and how neurostimulation methods enhance the ability to form accurate mnemonic representations.

## Author Contributions

AA and GI conceived the project and designed the task and environment. AA collected the behavioral and fMRI data. AA, GI, and AE wrote the manuscript and outlined the analysis pipeline and AA was responsible for analyzing the data.

## Conflict of Interest Statement

The authors declare that the research was conducted in the absence of any commercial or financial relationships that could be construed as a potential conflict of interest.

## Footnotes

1 https://unity3d.com

2 http://fsl.fmrib.ox.ac.uk/fsl/fslwiki/

3 https://github.com/stnava/ANTs

4 https://github.com/aestrivex/bctpy
